# Production of Mineral-Carbon Composites and Activated Carbons as a Method of Used Gear Oil, Ashes, and Low-Quality Brown Coals Management

**DOI:** 10.3390/molecules28196919

**Published:** 2023-10-03

**Authors:** Małgorzata Wiśniewska, Amanda Sadłowska, Karolina Herda, Teresa Urban, Piotr Nowicki

**Affiliations:** 1 Department of Radiochemistry and Environmental Chemistry, Institute of Chemical Sciences, Faculty of Chemistry, Maria Curie-Sklodowska University, M. Curie-Sklodowska Sq. 3, 20-031 Lublin, Poland; 2 Department of Applied Chemistry, Faculty of Chemistry, Adam Mickiewicz University in Poznań, Uniwersytetu Poznańskiego 8, 61-614 Poznań, Poland

**Keywords:** brown coal, ash, waste gear oil, mineral-carbon adsorbents, activated carbons, adsorption, water purification

## Abstract

The main objective of this study was to assess the usefulness of the low-quality brown coal, ash obtained as a result of its combustion, as well as used gear oil for the production of mineral-carbon adsorbents. The adsorbents were characterized in terms of textural parameters, acidic-basic character of the surface, mineral matter contribution to the structure, as well as their suitability for drinking water purification. Adsorption tests were carried out against two synthetic dyes—methylene blue and methyl orange. In order to understand the nature of the organic pollutants adsorption, the effect of the initial dye concentration, temperature, and pH of the system as well as the phase contact time were investigated. The obtained mineral-carbon composite and activated carbons significantly differed not only in terms of the elemental composition and chemical character of the surface (from slightly acidic to strongly alkaline), but also showed a very diverse degree of specific surface development (from 21 to 656 m^2^/g) and the type of porous structure generated (from micro/mesoporous to typically mesoporous). Adsorption tests showed that the efficiency of organic dye removal from aqueous solutions primarily depends on the type of the adsorbent and adsorbate applied, and, to a lesser extent, on the temperature and pH of the system. In turn, kinetic studies have shown that the sorption of dyes on such materials is consistent with a pseudo-second-order kinetics model, regardless of the type of adsorbed dye.

## 1. Introduction

Continuous technological development, human activity, as well as the increasing industrialization of the modern world cause a constant increase in the level of air and water pollution. The main threats to soils and surface waters are polluted domestic and industrial sewage, the use of large amounts of chemicals and the application of increasing amounts of pesticides in agriculture [1,2,3]. Water quality is getting increasingly worse, also due to the fact that water self-purification processes are insufficient [4]. It is a complicated process, which consists of physical, chemical, and biological stages, and its course is influenced by many factors, such as temperature, access to light, and the amount and type of pollutants introduced. Another problem in the environment is the growing human population, the development of cities and the progress of industry, which significantly affect air pollution [5,6]. Huge amounts of gases released into the atmosphere come from the exhausts of motor vehicles and the consumption of fossil fuels, the amount of which significantly increases from year to year. These gases primarily include nitrogen, sulfur, and carbon oxides, as well as volatile organic compounds [7].

Effective and successful wastewater and gas treatment is ensured, among others, by activated carbons through the use of the adsorption phenomenon. They mainly play the role of adsorbents, but also as catalysts and their carriers [8,9,10]. The problem of today’s world is also waste from the agricultural industry, which is generated on a very large scale [11]. Agricultural waste can be thermally and chemically treated to produce a wide range of valuable substances such as biofuels and biogas [12,13]. In order to minimize the amount of landfilled waste, a solution was found that constitutes a kind of alternative method of reusing it, and more specifically, the conversion of this waste into useful products—e.g., activated carbons. Materials such as sawdust, almond shells, hazelnut or coconut shells, olive stones, tree cones, and many others, which contain organic carbon in their structure, can be used for their production [14,15,16,17,18].

Brown coal is, qualitatively, a much worse fuel than hard coal. First of all, it is naturally very moist and easily absorbs water. This means that after extraction it contains about 30% water, and its calorific value is in the range of 5–10 MJ/kg, which is less than that for raw wood. After the drying process, it can reach approximately 20 MJ/kg, but it starts to crack. In addition, brown coal contains a lot of ash ~15–35 wt.% depending on the mine, and sometimes up to 50 wt.% (above this limit it is already road aggregate). It is also much more sulfated—it contains up to 5 wt.% of sulfur, which has a harmful effect on the natural environment. For this reason, a much better method of brown coal utilization, especially low-quality coal, is to subject it to a pyrolysis process and produce activated carbons. The same procedure can be applied to toxic and poisonous waste such as ashes coming from brown or hard coal combustion, as well as used engine or gear oils. Nearly 100 million tons of slags and ashes are annually produced in Europe. Their storage, recycling, and utilization pose many difficulties and are very expensive [19,20]. As a consequence of this, the illegal dumping of hazardous waste or its abandonment in forests, among other methods, has been increasingly reported. Therefore, the application of these waste materials as the precursors of activated carbons or carbon-mineral adsorbents is a very good method for their management and reuse [21,22,23,24]. These types of adsorbents can be successfully used for the adsorption of toxic gases of acidic character. Our previous research showed that adsorbents based on low-quality fossil coals, sewage sludge, and other waste materials with a high mineral content are very effective in removing nitrogen oxides and hydrogen sulfide from a gas stream [25,26,27].

Taking this into consideration, the main objective of the presented research was to determine the usefulness of low-quality brown coal, ashes from its combustion, as well as used gear oil for the production of mineral-carbon composites and activated carbons. The effect of the preparation procedure on the physicochemical properties and sorption capacity of the obtained materials was investigated. The obtained composite and activated carbons were characterized in terms of their textural properties, the acidic-basic character of the surface, the content of mineral admixtures in the structure, as well as in terms of suitability as adsorbents for the removal of organic pollutants from water. Adsorption tests were carried out against two toxic substances—methylene blue (cationic thiazine dye) and methyl orange (anionic azo dye). Adsorption kinetics, as well as the impact of the initial dye concentration, pH, and temperature of the solution on the efficiency of organic pollutants removal, were investigated.

## 2. Results and Discussion

### 2.1. Elemental Composition of the Precursors and Mineral-Carbon Adsorbents Prepared

According to the data collected in Table 1, the brown coal used for the study is characterized by a very high content of the mineral substance in the structure (slightly over 27 wt.%), which makes it a rather unsuitable material for energy purposes. Therefore, its application for the production of carbonaceous adsorbents could be an interesting and cost-effective alternative for its management. In the case of the second precursor, exhausted gear oil, the content of mineral admixtures is negligible (only 1.1 wt.%). The highest content of inorganic matter (94.9 wt.%) is characteristic of the mineral-carbon composite, which is of course a consequence of the use of ash as one of its components. Following on from the further analysis of the data presented in Table 1, the procedure of brown coal activation significantly affects the ash content in the activated carbons prepared. Sample BAp, obtained via the one-step direct physical activation of the precursor, fares much worse in this respect because it contains twice as much ash as the product of chemical activation (BAc). Too high ash content in the activated carbon structure has a negative impact on its sorption capacity because it constitutes unnecessary ballast, which makes it difficult for the adsorbate molecules to access the interior of the adsorbent pores.

The data summarized in Table 1 show that the brown coal selected for the study is also characterized by a low coalification degree and structure order, which is indicated by the high proportion of heteroatoms, especially oxygen. In the case of the exhausted gear oil, the carbon content is significantly higher and reaches almost 85 wt.%. The share of nitrogen and sulfur for both materials is at a comparable level. However, the waste oil is characterized by a significantly lower oxygen content and, at the same time, a twofold higher H^daf^ contribution than the brown coal. The lowest content of elemental carbon (not exceeding 3 wt.%) was found for the composite obtained as a result of the pyrolysis of ash and used gear oil mixture. The share of hydrogen and nitrogen in the structure of this material is also negligible. In turn, the sulfur contribution is surprisingly high and slightly exceeds the carbon content.

Analysis of the data presented in Table 1 shows that both of the procedures applied for brown coal activation significantly changed the percentage contribution of the elemental carbon and other elements in relation to the starting material. As a result of the thermochemical treatment of the precursor, the least stable fragments of its structure (rich in oxygen and hydrogen) are decomposed, which results in the progressive aromatization of the carbon matrix. These changes are much more pronounced for the BAc sample, obtained as a result of the chemical activation of brown coal with potassium carbonate at 700 °C.

### 2.2. Textural Parameters of the Mineral-Carbon Adsorbents Prepared

**Figure 1:**
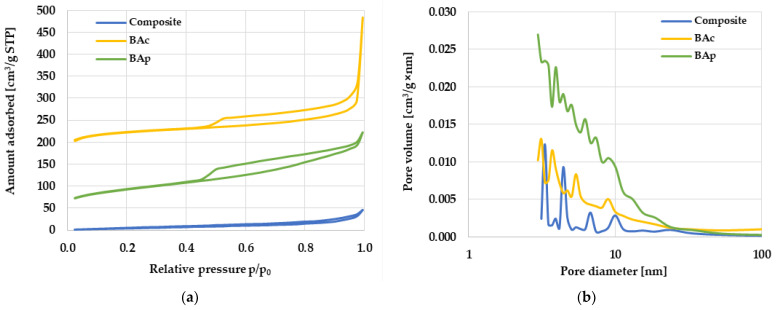
Low-temperature nitrogen adsorption/desorption isotherms (**a**) and pore size distribution (**b**) for the mineral-carbon composite and activated carbons.

In the next stage of the research, the textural parameters of the obtained mineral-carbon materials were determined, which are of significant importance in the context of practical application, especially in the field of adsorption. The data collected in Table 2 and Figure 1 reveal significant differences between the adsorbents depending on the procedure of preparation. The most desirable textural parameters (S_BET_ = 656 m^2^/g, V_T_ = 0.75 cm^3^/g) were found for sample BAc, obtained by means of chemical activation. Unfortunately, the one-step physical activation of brown coal turned out to be a much less effective solution in terms of porous structure development. The worst textural parameters (much more different from those obtained for the other samples) were mineral-carbon composite obtained via the pyrolysis of ash and used gear oil mixture. The surface area of this material was only 21 m^2^/g, and the total pore volume was 0.072 cm^3^/g. Such unfavorable textural parameters were most probably a consequence of the very high contribution of mineral matter in its structure (Table 1).

Further analysis of the data presented in Table 2 and Figure 1 shows that the porous structure of the composite mainly consists of mesopores and macropores, as evidenced by the complete absence of micropores and a very high value of the average pore diameter for this material, which is 13.37 nm. In turn, both activation products show a micro/mesoporous nature of the structure. The value of the average pore diameter for both activated carbons is about 4.5 nm, however, it should be emphasized that the chemically activated sample (BAc) has almost twice as many micropores in the total pore volume as the product of direct activation (BAp).

Isotherms of low-temperature nitrogen sorption recorded for the activated carbons (Figure 1a) resemble type I isotherm (according to IUPAC classification), confirming the significant share of micropores and small mesopores in the structure of these carbon materials. In the course of the isotherms, the presence of a hysteresis loop of the H4 type was noted, which indicated the occurrence of slit pores. In the case of the mineral-carbon composite, the shape of the isotherm was similar to type II, characteristic of non-porous or macroporous materials, in which there are very weak interactions between the adsorbate molecules and the adsorbent surface. In the course of this isotherm, a hysteresis loop similar to the H3 type was observed, indicating the presence of wedge/groove-shaped pores in the material. The diversification of the obtained mineral-carbon materials in terms of the type of generated pore structure was also confirmed by the course of the pore size distribution curves presented in Figure 1b. These data clearly indicate that, in the structure of both activated carbons, there is a much larger number of pores with sizes in the range of micropores and small mesopores (<25 nm) than for the composite.

**Figure 2:**
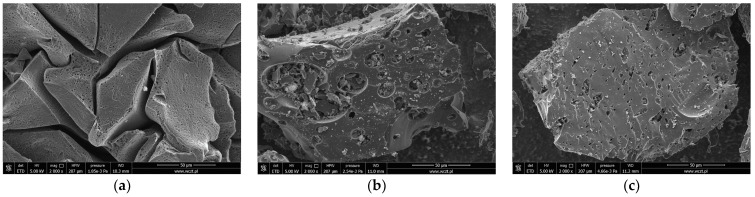
SEM images of the mineral-carbon composite (**a**), chemically (**b**) and physically (**c**) activated carbons.

The SEM images presented in Figure 2 also indicate significant textural and morphological differences between the mineral-carbon composite and both products of brown coal activation. All of the samples differ in terms of the size, shape, number, and distribution of slits or holes. The chemically activated carbon (BAc) has the most developed and diversified pore system, which confirms the previously discussed results of textural analysis. Moreover, in the case of both activated carbon samples, small particles of mineral substance (the brighter fragments) remaining in the pores can be observed.

### 2.3. Acidic—Basic Properties of the Mineral-Carbon Adsorbents Prepared

**Figure 3:**
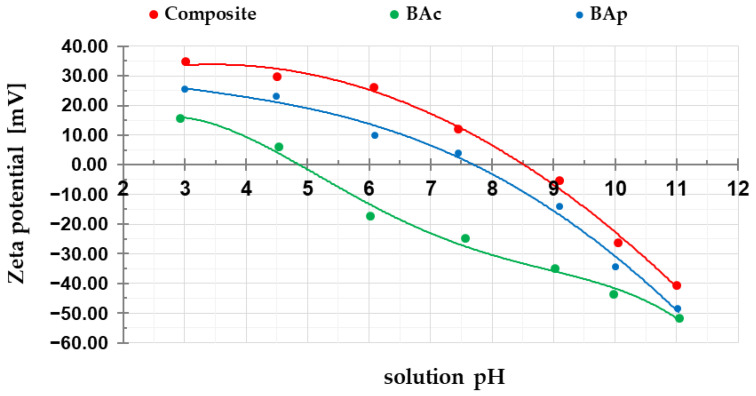
Changes in zeta potential of mineral-carbon composite and activated carbons as a function of solution pH.

To characterize the chemical nature of the mineral-carbon adsorbents’ surface, the pH value of their aqueous extracts, the content of functional groups of basic and acidic character, and zeta potential were determined. According to the data summarized in Table 3 and Figure 3, the obtained materials show completely different acidic–basic properties of the surface. The surface of the composite and the BAp-activated carbon has an alkaline character. This is evidenced by the high pH values of the aqueous extracts (ranging from 9.62 to 11.80) and the high content of basic functional groups (3.10 and 4.08 mmol/g, respectively). This is most probably a consequence of the very high ash content in the structure of these materials (Table 1). In turn, the BAc sample (obtained by chemical activation of brown coal with potassium carbonate and characterized by the lowest contribution of mineral substance, i.e., 17.0 wt.%) shows a slightly acidic character of the surface (pH = 6.11).

The type and amount of functional species formed on the surface of individual adsorbents significantly depend on the variant of thermochemical treatment used to obtain them. Both the composite and the product of direct activation are characterized by a definite predominance of basic functional groups, while the sample obtained as a result of chemical activation contains almost twice as many acidic groups as basic groups. Such a significant predominance of basic functional groups over acidic ones can be caused by the high content of mineral substances in the structure of mineral-carbon adsorbents as well as by the use of carbon dioxide in the direct activation procedure, which together with a high activation temperature (800 °C) favors the generation of the basic functional groups.

According to the data presented in Figure 3 (curves of zeta potential changes as a function of solution pH), the isoelectric points (pH_iep_) are located at pH values of: 8.5 for the mineral-carbon composite, 7.7 for physically activated carbon, and 4.8 for the chemically activated sample. As seen, these pH_iep_ values differ quite significantly from the pH of aqueous extracts (Table 3), especially in the case of the composite sample. As is commonly known, the value of this parameter is important from the adsorption point of view. Above the pH_iep_, the charge of the diffusion part of the electrical double layer is negative, which favors the adsorption of cationic impurities, e.g., methylene blue. In turn, below the pH_iep_, the positively charged surface of the adsorbent favorably interacts with anionic substances, e.g., methyl orange

### 2.4. Sorption Performance of the Mineral-Carbon Adsorbents in Relation to Organic Dyes

**Figure 4:**
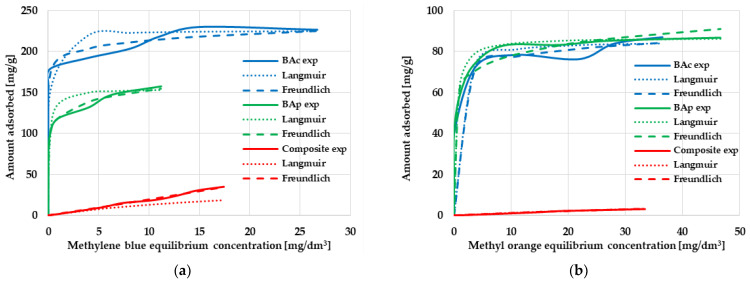
The equilibrium isotherms of methylene blue (**a**) and methyl orange (**b**) adsorption on the mineral-carbon composite and activated carbons.

In order to determine the sorption abilities of the mineral-carbon adsorbents and to evaluate their suitability for the removal of organic impurities from the aqueous solutions, two adsorbates of various molecular sizes and chemical character were applied; i.e., methylene blue (Mb) and methyl orange (Mo). The results of the adsorption tests are presented in Figure 4 and Table 4.

According to the data displayed in Figure 4, the obtained mineral-carbon composite and activated carbons are characterized by a very diverse sorption capacity towards individual organic dyes. However, it should be clearly emphasized that much better results were obtained during the adsorption of methylene blue from the aqueous solutions. As seen, the adsorption efficiency significantly increases with increasing the initial concentration of Mb and Mo in the solution. It is most likely related to the fact that, at low organic dye concentrations, the adsorption of this type of pollutant on the surface of mineral-carbon adsorbents is completely random. At higher concentrations of methylene blue and methyl orange, all active centers present on the adsorbents’ surface are filled up and, as a consequence, a state of full saturation is reached.

The most effective adsorbent in relation to methylene blue turned out to be the BAc sample obtained as a result of the chemical activation of brown coal, which was able to adsorb 233.4 mg of dye per gram of the solid. The composite and product of direct physical activation were able to adsorb only 34.8 and 156.4 mg of Mb, respectively, which was most probably related to their less favorable textural parameters. A completely different situation was observed in the case of methyl orange adsorption, where the sorption capacity of both activated carbons was about 85 mg/g, with a slight predominance of the BAp sample. The almost identical value of q_exp_ (despite a significant difference in the textural parameters of both materials) suggested that the effectiveness of Mo adsorption may be conditioned by the surface chemistry of the adsorbents, as well as the share of mineral admixtures in their structure. The mineral-carbon composite was definitely less favorable in this respect, and its sorption capacity was only 3.1 mg/g.

Further analysis of the data presented in Table 4 indicates that the adsorption of both organic dyes on the obtained activated carbons may proceed according to the mechanism proposed by Langmuir; i.e., with the formation of an adsorbate monolayer on the adsorbent surface. This is suggested by the very high values of the coefficient of determination R^2^ (ranging from 0.9939 to 0.9997), as well as by the comparable values of the experimental (q_exp_) and calculated (q_max_) sorption capacities. However, analysis of the course of the isotherms shown in Figure 4 indicates that the methylene blue and methyl orange adsorption mechanisms are much more complicated. As seen, neither the Langmuir isotherm nor the Freundlich one accurately describes the experimental data over the entire range. In the case of the mineral-carbon composite, the Freundlich isotherm fits the experimental data better than the Langmuir one, which may indicate a multilayer adsorption process on a heterogeneous surface as well as interactions between adsorbate molecules. However, a precise determination of the mechanism of the adsorption of cationic and anionic dyes on mineral-carbon materials requires further research.

**Figure 5:**
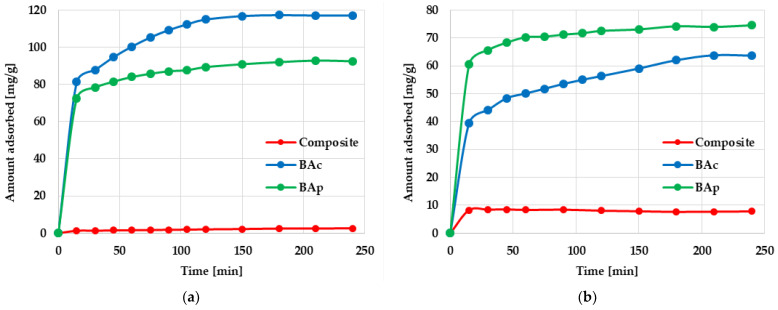
Effect of phase contact time on methylene blue (**a**) and methyl orange (**b**) adsorption on the mineral-carbon composite and activated carbons.

The next stage of the research was to determine the kinetic parameters of the adsorption process. The effect of the contact time on methylene blue and methyl orange adsorption on mineral-carbon adsorbents is shown in Figure 5. According to these data, the kinetics of both dyes’ adsorption included two main stages: (1) very fast adsorption of Mb and Mo molecules, which significantly affected the equilibrium dye’s uptake; (2) gradual and slow adsorption of dyes leading to full saturation and an equilibrium state.

Pseudo-first-order (PFO) and pseudo-second-order (PSO) kinetics models were used for the analysis of experimental data. According to the results presented in Table 5, the experimental data do not correspond to the PFO model, regardless of the type of adsorbed dye. Values of the coefficient of determination range from 0.6920 to 0.9659, and the calculated sorption capacities (q_cal_) significantly differ from those experimentally determined (q_exp_). A much better fit of the experimental data was obtained using the pseudo-second-order kinetics model. In this case, the value of the R^2^ coefficient for all adsorbent-adsorbate systems change in the very narrow range of 0.9725–0.9999, whereas the differences between the values of q_cal_ and q_exp_ are insignificant.

**Figure 6:**
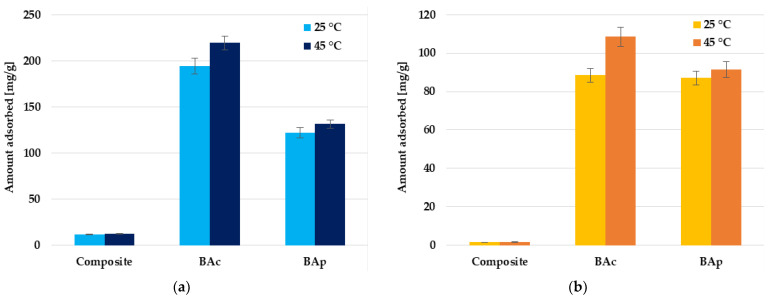
Effect of temperature on methylene blue (**a**) and methyl orange (**b**) adsorption on the mineral-carbon composite and activated carbons.

According to the data displayed in Figure 6, the efficiency of organic dye removal from aqueous solutions depends on the temperature of the mineral-carbon adsorbent-adsorbate system. In the case of methylene blue and methyl orange removal, the increase in temperature from 25 °C to 45 °C results in a significant improvement of the adsorption capacity, especially for the BAc sample (by 13 and 23%, respectively). The diffusion of the Mb or Mo molecules is affected by the temperature, which leads to a higher mass transfer rate from the bulk to the boundary layer around the surface of the mineral-carbon adsorbent particles. The increase in the adsorption capacity with increasing temperature of the adsorbent-organic dye aqueous solution system may suggest that the reaction between the mineral-carbon adsorbents and methylene blue or methyl orange molecules is endothermic, and the adsorption process is chemical in nature.

**Figure 7:**
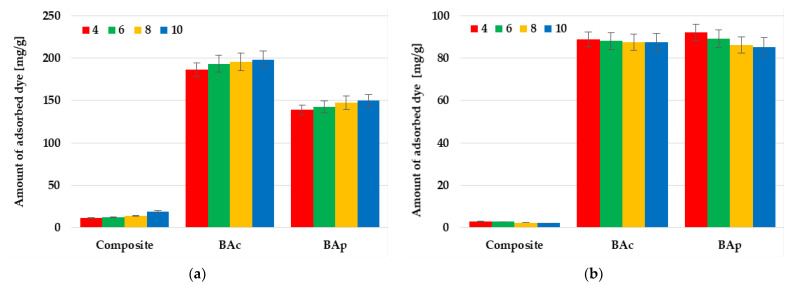
Effect of solution pH on methylene blue (**a**) and methyl orange (**b**) adsorption on the mineral-carbon composite and activated carbons.

Based on the data presented in Figure 7, it can be concluded that another parameter that significantly affects the adsorption capacity of the tested mineral-carbon materials toward individual dyes is the pH of the solution. For each of the tested adsorbent-adsorbate systems, the influence of pH is much greater in the case of methylene blue removal. Each of the adsorbents shows the highest efficiency of adsorption of this dye at pH 10. The difference in sorption capacity noted at pH 4 and 10 is equal to 11.9, 10.7, and 7.3 mg/g for BAc, BAp, and the mineral-carbon composite, respectively. This is a consequence of the fact that, in solutions of an alkaline nature, the functional groups present in the carbonaceous material structure are deprotonated and a negative charge appears on the adsorbent surface. This enhances the electrostatic attraction between the methylene blue cations and the negatively charged carbon adsorbent surface.

A completely opposite trend in changes was observed in the case of methyl orange adsorption, where the highest sorption capacities were observed at a pH equal to 4. In acidic solutions, the surface functional groups were protonated, which favored the interaction of the positively charged adsorbent surface with anionic dye molecules. However, the differences between the sorption capacity reached at pH 4 and 10 were much smaller and were equal to 8.6 mg/g for physically activated carbon, 1.4 mg/g for the chemically activated sample, and only 0.9 mg/g in the case of the composite.

Following on from the analysis of the data collected in Table 6, the obtained mineral-carbons adsorbents performed quite well in terms of organic dye removal from the aqueous solutions when compared with different adsorbents described in the literature of the subject; therefore, after optimizing the procedure for their production, they can be successfully used for wastewater or drinking water treatment. The product of the chemical activation of low-quality brown coal is particularly good in this regard.

## 3. Materials and Methods

### 3.1. Mineral-Carbon Adsorbents Preparation

**Figure 8:**
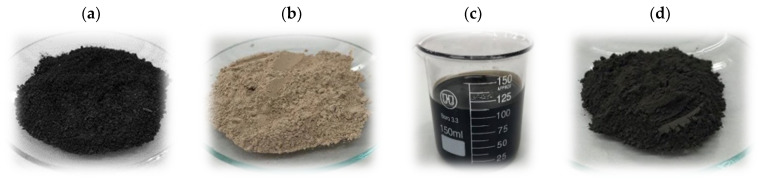
Precursors used for the study: low quality brown coal (**a**), ash (**b**), waste gear oil (**c**), as well as the mineral-carbon composite produced (**d**).

One of the precursors was brown coal from the KWB Konin Mine (Tomisławice opencast, Poland) with a grain size below 1.6 mm (Figure 8a). In order to produce the ash (used as the second precursor), several portions of the starting brown coal (each about 10 g) were placed in porcelain crucibles and burned in a muffle microwave furnace (Phoenix model, CEM Corporation, Matthews, IL, USA) at a temperature of 850 °C for 1 h. The ash obtained in this way was ground in a porcelain mortar to homogenize the sample (Figure 8b). A third of the precursors used for the production of mineral-carbon adsorbents was exhausted Mobilube™ 1 SHC 75W-90 gear oil (ExxonMobil Poland, Warszawa, Poland; Figure 8c).

At the beginning, the ash was physically mixed with the exhausted gear oil, at a weight ratio equal to 1:2. After 20 h of the impregnation stage (with occasional mechanical stirring at a temperature of 25 °C), the mixture was placed in a quartz boat and subjected to thermal treatment in a resistance laboratory furnace (Thermo Fisher Scientific Inc., Waltham, MA, USA) according to the following procedure:

(step 1) heating of the sample in a nitrogen atmosphere (N_2_ flow 10 dm^3^/h), from room temperature to 200 °C (heating rate 5 °C/min);
(step 2) annealing of the sample at 200 °C for 0.5 h;
(step 3) heating of the sample to a temperature of 500 °C (heating rate 5 °C/min);
(step 4) annealing of the sample at 500 °C for 1 h.

Finally, the sample was cooled down and ground in a porcelain mortar (Figure 8d).

Another portion of the starting brown coal (B) was subjected to a direct physical activation process (Ap). This process was carried out in a one-heating-zone laboratory furnace, equipped with a quartz tubular reactor (Thermo Fisher Scientific Inc., Waltham, MA, USA). Approximately 10 g of the precursor was placed in the nickel boat and subjected to thermal treatment under a carbon dioxide atmosphere (technical CO_2_ 2.8, Linde Gaz Polska, Kościan, Poland; flow rate 15 dm^3^/h). The boat with the sample was placed in the furnace preheated to a temperature of 800 °C and annealed under these conditions for a period of 45 min. Finally, the sample was taken out from the hot zone of the furnace and cooled down to room temperature (using an external fan).

The remaining part of the starting brown coal was chemically activated (Ac) according to the following procedure: at the beginning, the precursor was impregnated with K_2_CO_3_ solution (Avantor Performance Materials, Gliwice, Poland) at a brown coal/activating agent weight ratio of 1:2. After the impregnation stage (24 h at room temperature, with occasional stirring), the sample was dried at 110 °C to evaporate the water, placed into a quartz boat, and subjected to thermal treatment in a one-heating-zone laboratory furnace, equipped with a quartz tubular reactor (Czylok, Jastrzębie-Zdrój, Poland). Thermochemical treatment of the impregnated precursor was carried out under a nitrogen atmosphere (technical nitrogen 4.0, Linde Gaz Poland, Kościan, Poland; flow rate 20 dm^3^/h) and consisted of two main stages:

(step 1) heating of the sample from room temperature to a final activation temperature of 700 °C (heating rate 10 °C/min),
(step 2) annealing of the sample at 700 °C for 0.5 h.

Finally, the sample was cooled down to room temperature, subjected to a two-stage post-activation washing procedure with hot 5% hydrochloric acid (Avantor Performance Materials, Gliwice, Poland) and boiling distilled water, respectively, and dried at 110 °C to constant mass. Both variants of activation were also applied to the previously obtained mineral-carbon composite. Unfortunately, the effects of this action were very unsatisfactory—the yield of the process at the level of 10 wt.% (in the case of chemical activation) as well as a significant deterioration of the textural parameters (in the case of physical activation) undermined the sense of such a procedure, both for economic and ecological reasons. Therefore, this research path was not continued.

### 3.2. Physicochemical Characterization of the Precursors and Mineral-Carbon Adsorbents

The elemental analysis of the precursors as well as products of their thermochemical conversion was performed using a CHNS Vario EL III instrument (Elementar Analysensysteme GmbH, Langenselbold, Germany). The total ash content for all the materials under investigation was determined according to the ISO 1171:2002 Standard, using a microwave muffle furnace (CEM Corporation, Matthews, IL, USA).

Textural characterization of the mineral-carbon adsorbents was based on the N_2_ adsorption-desorption isotherms, measured at –196 °C on an Autosorb iQ sorptometer, provided by Quantachrome Instruments (Boynton Beach, FL, USA). Before isotherm measurement, the samples were outgassed at 300 °C (under vacuum). The specific surface area of the materials was determined in the range of relative pressure (p/p_0_), changing from 0.05 to 0.30. The total pore volume was calculated by converting the liquid nitrogen amount adsorbed at p/p_0_ ~0.99. The pore size distribution was calculated from the adsorption branches of isotherms using the Barrett-Joyner-Halenda (BJH) method. The *t-plot* method was applied to determine the micropore volume and micropore surface area.

Morphologies of the mineral-carbon composite and activated carbons were analyzed using a Quanta 250 FEG-SEM high-resolution electron microscope, provided by FEI (Waltham, MA, USA).

Electrophoretic mobility (*u_e_*) measurements, enabling the determination of the zeta potential (ζ) and the isoelectric point (iep) of carbonaceous adsorbents particles, were performed using a Zetameter Nano ZS (Malvern Instruments, Cambridge, UK). Based on Henry’s equation [40], the electrokinetic potential values of the examined systems were calculated. The suspensions were prepared by the addition of 0.05 g of the solid to 0.2 dm^3^ of doubly distilled water. Such a prepared system was sonicated (Ultrasound XL 2020, Misonix, Markham, ON, Canada) for 3 min and divided into several parts, differing in terms of pH value (changing in the range 3–11) and adjusted using a PHM 240 pH meter (Radiometer, Apeldoorn, The Netherlands). Then, the electrophoretic mobility of each sample was measured using a dip cell in cycles of five repetitions.

The content of the surface functional groups was determined according to the Boehm titration method, described in detail in our previous paper [41]. To specify the chemical nature of the surface of the mineral-carbon adsorbents, the pH value of their water extracts was also determined, using the procedure described in [10].

### 3.3. Adsorption of Methylene Blue and Methyl Orange

In order to determine the usefulness of the mineral-carbon materials for the removal of organic pollutants from the aqueous solution, adsorption tests on two synthetic dyes, methylene blue (Mb) and methyl orange (Mo), were carried out. The following procedure was applied for each of the adsorbents and adsorbates under investigation: a series of the adsorbent samples in equal portions of 0.025 g were mixed with 0.05 dm^3^ of the Mb or Mo aqueous solutions (Avantor Performance Materials, Gliwice, Poland) with initial concentrations varying in the range of 5–200 mg/dm^3^ and magnetically stirred (150 rpm, 24 h, temperature of 22 ± 1 °C) to reach the equilibrium state. After that, the solids were separated by centrifugation (FC5515 microcentrifuge, OHAUS, Parsippany, NJ, USA; 15,000 rpm, for 10 min) and the clear solutions obtained were subjected to spectrophotometric analysis. The Mb and Mo concentrations in the solution before and after the adsorption tests were established using a double beam UV–Vis spectrophotometer Cary 100 Bio (Agilent, Santa Clara, CA, USA) at wavelengths of: 664 nm (for methylene blue) and 464 nm (for methyl orange), using the previously prepared calibration curves. Distilled water was applied as a reference sample in both cases.

The amount of particular dye adsorbed at the equilibrium state (q_e_, mg/g) was calculated according to the following Formula (1):

(1) $$q_{e}=\frac{{∆c}_{dye}\cdot V_{aq\;sol}}{m_{ads}}$$

where Δc_dye_ is the difference in the organic dye concentration before and after adsorption [mg/dm^3^], V_aq sol_ is the volume of the organic dye aqueous solution used for the test [dm^3^], m_ads_ is the mass of mineral-carbon adsorbent used [g].

The experimental data were further analyzed according to Langmuir and Freundlich adsorption isotherm models [42].

The influence of temperature on the efficiency of the adsorption of the abovementioned organic compounds from aqueous solution was determined according to the following procedure: a portion of 0.025 g of each mineral-carbon adsorbent was placed in a flat-bottomed flask and mixed with 0.05 dm^3^ of Mb or Mo solution. Such prepared suspensions were placed on a Unimax 1010 shaker (equipped with an incubator, Heidolph Instruments GmbH & Co. KG, Schwabach, Germany) and shaken for 24 h at temperatures of 25 °C and 45 °C. The further measurement procedure was the same as before.

The pH effect on the removal efficiency Mb and Mo was studied according to the following procedure: a portion of 0.025 g of the mineral-carbon composite or activated carbon sample was shaken with 0.05 dm^3^ of dye solution at a concentration of 20 mg/dm^3^ (in the case of the composite, 80 mg/dm^3^ in the case of BAp sample, and 100 mg/dm^3^ in the case of the BAc sample) for 6 h, at a pH changing in the range 4.0–10.0. After shaking, the suspension was centrifuged and the final concentration of dye was determined as described above. The solution pH before the experiments was adjusted by adding appropriate amounts of 0.1 M HCl or 0.1 M NaOH (Avantor Performance Materials, Gliwice, Poland).

The procedure for the kinetic experiments was analogous to that applied for the equilibrium tests. The suspension was magnetically stirred (200 rpm) for 4 h, at a temperature of 22 ± 1 °C. Samples of the solution were collected at intervals of 15 min and subjected to Mb or Mo concentration measurements. The amount of particular dye adsorbed at time t (q_t_, mg/g) was calculated according to the following Formula (2):

(2) $$q_{t}=\frac{(c_{0}-c_{t})\cdot V_{aq\;sol}}{m_{ads}}$$

where c_0_ is the initial concentration of dye in the aqueous solution (mg/dm^3^), c_t_ is the dye concentration after time t [mg/dm^3^], V_aq sol_ is the volume of the dye solution (dm^3^), and m_ads_ is the mass of adsorbent used (g). Experimental data were analyzed using the pseudo-first-order kinetics model (PFO) proposed by Lagergren and the pseudo-second-order kinetics model (PSO) proposed by Ho and McKay [43].

## 4. Conclusions

The conducted studies showed that materials such as low-quality brown coals with a high mineral matter content, ashes obtained as a result of their combustion, as well as used gear oil can be successfully used for the production of new mineral-carbon adsorbents, characterized by very interesting physicochemical properties. At the current stage of research, some of the prepared composites are characterized by a rather poorly developed porous structure and specific surface area, which may result in the rather low efficiency of organic pollutant removal from aqueous solutions. Nevertheless, there are no literature reports on the use of these hazardous precursors to produce this type of adsorbent. It is therefore necessary to further improve their production procedure, which is extremely important for the management of toxic and burdensome waste such as ashes and used gear oils. The most promising material in this regard is activated carbon obtained via the chemical activation of low-quality brown coal, which shows sorption abilities similar to those of commercial products. The conducted adsorption tests proved that the efficiency of organic pollutant removal from aqueous solutions significantly increases with the increase in initial dye concentration as well as the temperature of the adsorbent-adsorbate system. With the increasing alkalinity of the solution, the effectiveness of methylene blue adsorption on the materials obtained increases, whereas in the case of methyl orange removal, the opposite tendency is observed. Kinetic studies have shown that the adsorption of methylene blue and methyl orange on this type of material takes place in accordance with the pseudo-second-order kinetics model, regardless of the type of adsorbed dye. The obtained mineral-carbon sorbents, due to their very interesting physicochemical properties, especially the strongly alkaline nature of the surface, can also be potentially used in the adsorption of heavy metal ions, acidic gas pollutants (e.g., NO_2_ or H_2_S), or as catalysts or catalyst supports. However, this requires the optimization of their production procedure and the performance of appropriate adsorption or catalytic tests.

## Figures and Tables

**Table 1:** Elemental composition of the precursors, mineral-carbon composite, and activated carbons [wt.%].

Sample	Ash	Nitrogen^daf^	Carbon^daf^	Hydrogen^daf^	Sulphur^daf^	Oxygen^diff^
Brown coal	27.1 ± 1.27	0.5 ± 0.02	57.3 ± 0.28	5.6 ± 0.08	1.2 ± 0.03	35.4 ± 0.49
Waste gear oil	1.1 ± 0.08	0.4 ± 0.01	84.7 ± 0.92	11.3 ± 0.21	1.0 ± 0.04	2.6 ± 0.13
Composite	94.9 ± 0.76	0.1 ± 0.03	3.0 ± 0.18	0.2 ± 0.06	3.5 ± 0.10	93.2 * ± 0.71
BAc	36.6 ± 0.72	0.4 ± 0.02	82.6 ± 0.57	1.3 ± 0.07	0.4 ± 0.01	15.3 ± 0.16
BAp	17.0 ± 0.78	0.6 ± 0.04	75.9 ± 0.35	0.7 ± 0.03	2.8 ± 0.13	20.0 ± 0.65

^daf^—dry-ash-free basis; ^diff^—calculated by difference; * including ash.

**Table 2:** Textural parameters of the mineral-carbon adsorbents.

Sample	Surface Area ^1^ [m^2^/g]	Micropore Area [m^2^/g]	Total Pore Volume [cm^3^/g]	Micropore Contribution	Mean Pore Size [nm]
Composite	21	-	0.072	-	13.37
BAc	656	588	0.750	0.43	4.57
BAp	301	159	0.345	0.26	4.58

^1^ method error in the range from 2 to 5%.

**Table 3:** Acidic-basic properties of the mineral-carbon composite and activated carbons.

Sample	pH of Aqueous Extracts	Basic Groups Content [mmol/g]	Acidic Groups Content [mmol/g]	Total Content of Surface Groups [mmol/g]
Composite	11.80 ± 0.07	3.10 ± 0.10	0.48 ± 0.04	3.58 ± 0.08
BAc	6.11 ± 0.04	0.57 ± 0.03	0.99 ± 0.03	1.56 ± 0.04
BAp	9.62 ± 0.11	4.08 ± 0.14	0.25 ± 0.06	4.33 ± 0.04

**Table 4:** Langmuir/Freundlich parameters of the equilibrium isotherms of methylene blue and methyl orange adsorption on the mineral-carbon composite and activated carbons.

Sample	q_exp_	Langmuir Model			Freundlich Model		
		q_max_	K_L_	R^2^	K_F_	1/n	R^2^
Methylene blue							
Composite	34.8	45.1	0.04	0.4585	1.79	1.037	0.9856
BAc	233.3	234.7	4.06	0.9987	186.90	0.060	0.8869
BAp	156.4	154.1	7.55	0.9960	118.44	0.109	0.9811
Methyl orange							
Composite	3.1	3.3	0.15	0.7728	5.35	0.813	0.9647
BAc	83.2	85.3	1.61	0.9939	65.16	0.071	0.7201
BAp	85.3	86.7	2.91	0.9997	62.03	0.100	0.8719

q_exp_—experimental adsorption capacity [mg/g], q_max_—the maximum adsorption capacity [mg/g], K_L_—the Langmuir adsorption equilibrium constant [dm^3^/mg], K_F_—the Freundlich equilibrium constant [mg/g (mg/dm^3^)^1/n^], 1/n—the intensity of adsorption, R^2^—the determination coefficients.

**Table 5:** Kinetic parameters for methylene blue and methyl orange adsorption on the mineral-carbon composite and activated carbons.

Sample	q_exp_	Pseudo-First Order			Pseudo-Second Order		
		k_1_	R^2^	q_cal_	k_2_	R^2^	q_cal_
Methylene blue							
Composite	2.698	0.013	0.8978	2.388	0.007	0.9725	3.092
BAc	117.821	0.027	0.9386	76.243	0.001	0.9975	126.852
BAp	93.407	0.018	0.9659	30.775	0.001	0.9997	96.154
Methyl orange							
Composite	2.988	0.005	0.9062	0.462	0.023	0.9988	3.435
BAc	67.537	0.012	0.9577	37.394	0.001	0.9954	71.429
BAp	74.519	0.020	0.6920	18.450	0.003	0.9999	75.758

q_exp_—experimental adsorption capacity [mg/g], q_cal_—calculated adsorption capacity [mg/g], k_1_—the pseudo-first-order adsorption rate constant [1/min], k_2_—the pseudo-second-order adsorption rate constant [g/(mg∙min)], R^2^—the determination coefficients.

**Table 6:** Adsorption capacities towards methylene blue and methyl orange for various adsorbents.

Adsorbent	Adsorbed Amount [mg/g]	Reference
Methylene blue		
Composite	35	This study
BAp	156	This study
BAc	233	This study
Activated carbon derived from lignocellulosic wastes (physically activated by H_2_O-steam)	149	[28]
Mesoporous-activated carbon from low-rank coal (microwave-induced KOH-activation method)	492	[29]
Mesoporous-activated carbon from agricultural wastes including oil palm frond and palm kernel shell (microwave radiation-assisted K_2_CO_3_ activation)	332	[30]
Zeolite–activated carbon composite from oil palm ash (chemical activation with NaOH and hydrothermal treatment)	144	[31]
Porous graphene–carbon nanotubes composite (hydrothermal reaction)	232	[32]
Hydroxyapatite and hydroxypropyl methylcellulose-based nanocomposite (dissolution/reprecipitation method)	52	[33]
Methyl orange		
Composite	3	This study
BAp	85	This study
BAc	83	This study
Nitrogen-rich biomass-derived carbon adsorbent made from Enteromorpha	270	[34]
Biochars produced from agro-waste and invasive plants: wattle bark, mimosa, coffee husks	12	[35]
Metal ion (Fe^3+^, Mg^2+^, Ca^2+^, and Na^+^) modified biomass of waste beer yeast	23–91	[36]
Magnetic clay-biochar composite	63	[37]
Imidazolate-zeolite frameworks-11	179	[38]
Alkali-activated multiwalled carbon nanotubes	149	[39]

## Data Availability

Data are contained within the article.

## Sample Availability

Samples of the mineral-carbon adsorbents are available from the authors upon reasonable request.
